# Extracts from the Records of the Suffolk District Medical Society

**Published:** 1856-05

**Authors:** 


*Extracts from the Records of the Suffolk District Medical Society.*
J. B. Alley, M. D., Secretary.

*Jan. 26. Abdominal Abscess.* Dr. Channing reported the following case:

The patient, a man æt. 35, was seized with severe pain in the right iliac region, accompanied by a tremor, which appeared to be situated nearly on a level with the umbilicus. This was followed by night-sweats, nausea, much emaciation, and embarrassment of the bowels and bladder. An evacuation of pus and blood from the rectum occurred, and the tumor subsided: the last discharge of matter occurring in the course of two years and six months. After an exposure late at night on a steamer, he had a distinct chill, and a tumor formed in the left iliac region, accompanied with severe pain, and occupying as large a surface as the other. This was opened by Dr. Townsend, sen., and discharged a quantity of pus having a fæcal smell. The discharge continued for some weeks, with much prostration of strength, and he lost forty pounds in weight. Upon the subsidence of the second iliac enlargement, he was seized with pain in the right hypochondrium at top of right shoulder. This was soon followed by an enlargement which gradually extended downward to a level with the umbilicus, and upward to so great a degree as to project the ribs and fill the auscultatory space. There wa- dullness on percussion, but an attentive ear could detect the ordinary vesicus lar murmur, and no broncophony. After riding out one day, he coughed the same night, and raised a quantity of fœtid and highly offensive matter, and continued to do so for some weeks. This occurred at the sea shore, whither he had gone on account of the heat of the weather, and frequent night-sweats. The sputa did not possess a cadaveric smell, nor was the breath at all tainted as in gangrene of the lungs. Here, then, were three consecutive attacks of disease, each terminating in suppuration. Ten days ago, he was seized with severe pain in the abdomen, accompanied with a chill, but the difficulty subsided, and he is now well, complaining only of an occasional feeling as if ligatures were drawn around the organs.

Dr. Bowditch reported the following case:

The patient, a woman, two years since had an enormous effusion into the cavity of the chest, threatening immediate death. Paracentecis thoracis was performed twice, and a large quantity of fluid drawn off, with great relief. The lung expanded, but still an extreme dullness on percussion remained, and the attending physician diagnosed "tubercular consumption." Dr. B. thought that it would probably end in phthisis, but as he could not find any positive physical signs, except those of chronic pleurisy, he refrained from expressing a decided opinion in the case. After this, the patient slowly improved, resumed her household duties, and considered herself as improving in health, her cough having almost entirely subsided. A few weeks since, she was suddenly seized with acute inflammation within the pelvis, and died. Not a trace of tubercle was found in either lung, but in the cavity of the pleura, on one side, a pint of pus, the lung on that side being much increased in density by the compression of the fluid effused.

Dr. Bowditch alluded to the case of a child, aged 7, who, after having been thoroughly chilled, was seized with violent pain in the right side, followed by hemiplegia. He recovered from the paralysis, but every year, during cold weather, has violent pain of a neuralgic character, for three or four weeks, extending down the right side, and accompanied with constant twitching of the muscles of the face. The pulse was strong and firm; mind clear. Digitalis was exhibited, combined with opium, and the child ordered to be sent to a warm climate.

Dr. C. Page inquired what effect veratrine would exert?

Dr. B. replied that he thought it might be a valuable adjuvant in the treatment.

Dr. Buck alluded to the case of a woman, who, after a natural, easy labor, in which the pains were regular, and the child was not thrust forcibly into the world, had, ever since its birth, been unable to retain her fæces. No laceration of the perineum had occurred. Astringent injections had been tried without effect.

Dec. 29. Dr. Keep made some remarks upon the mechanical influence of the teeth in one jaw upon the relative position of those in the opposing jaw. He exhibited a double cast of the teeth of a lad of 14 years, in which the six front lower teeth were forced forward by the strong, can ne teeth o the upper jaw coming between the canines and bicuspids of both sides in the lower jaw, making considerable space between them, which was wholly unavailing to the displaced teeth by reason of the wedge-like action of the upper canines. In this case, the upper and lower front teeth articulated on their points, or cutting edges, which will no doubt cause them rapidly to wear down, and eventually to be called double teeth. He also remarked that cases frequently occur where the oblique angle of the articulating surfaces of two or more teeth to the plane of the jaw, was a cause of suffering, and premature loss of the teeth by dislocation. He had found the removal of a prominent point, by filing or cutting from one or both of the teeth which impinge thus unfavorably, of essential service.

In answer to a question from Dr. J. B. S. Jackson, Dr. Keep stated, that decay did not usually follow the abrasion of enamel on the grinding surfaces of an adult tooth, though in other situations, as between contiguous teeth, the integrity of the enamel was very important.

Dr. Bowditch inquired what had been Dr. Keep's experience as to the effect on the health, where whole sets of artificial teeth had been worn.

Dr. Keep called to mind several cases where persons had greatly improved in health, spirits and appearance, after being supplied with new teeth.

Dr. Bowditch remarked that he made the inquiry because he had recently had a patient who thought that his appetite was impaired by the use of false teeth.

Dr. Gould inquired of Dr. Keep if he had seen any cases where the lateral incisors were not present.

Dr. K. replied that he had, and that a patient, aged 55, had called on him that very day, who had cut a canine tooth within two years.

Dr. Homans, sen., added that he knew of a similar case, where the patient, a female, had cut a good sound canine tooth at the age of 64 years.

Dr. Jackson alluded to a case of salivation, caused by minute doses of blue mass, which might have been avoided if due regard had been paid to the idiosyncrasy of the patient.

Dr. Homans, sen., mentioned a case of salivation occurring in a child, affected with bronchitis, where the quantity of calomel exhibited was very small and the bowels were freely opened.

Dr. Jackson reported a case of a child of eighteen months, who had a propensity to swallow every article which it could take in its hand, and put into its mouth. In the course of three or four days it had passed from its bowels several pins and buttons, and also a cuff-pin, which had been voided with the pin unclasped.—*Boston Med. & Surg. Journal.*

